# A comparison of univariate and meta-analytic structural equation modeling approaches to reliability generalization applied to the Maslach Burnout Inventory

**DOI:** 10.3389/fpsyg.2024.1383619

**Published:** 2024-05-03

**Authors:** Raimundo Aguayo-Estremera, Gustavo R. Cañadas-De la Fuente, Tania Ariza, Elena Ortega-Campos, José Luis Gómez-Urquiza, José Luís Romero-Béjar, Emilia I. De la Fuente-Solana

**Affiliations:** ^1^Department of Psychobiology and Methodology in Behavioral Science, Universidad Complutense de Madrid, Madrid, Spain; ^2^Department of Didactic of Mathematics, Faculty of Education Science, University of Granada, Granada, Spain; ^3^Department of Educational Psychology and Psychobiology, Faculty of Education, Universidad Internacional de La Rioja, Logroño, Spain; ^4^CEINSA-UAL, University of Almería, Almería, Spain; ^5^Faculty of Health Sciences, University of Granada, Ceuta, Spain; ^6^Department of Statistics and Operations Research, University of Granada, Granada, Spain; ^7^Instituto de Investigación Biosanitaria (ibs. GRANADA), Granada, Spain; ^8^Institute of Mathematics, University of Granada (IMAG), Granada, Spain; ^9^Brain, Mind and Behavior Research Center (CIMCYC), University of Granada, Granada, Spain

**Keywords:** MBI, MASEM, meta-analysis, reliability generalization, burnout

## Abstract

**Introduction:**

Reliability is a property of tests scores that varies from sample to sample. One way of generalizing reliability of a test is to perform a meta-analysis on some reliability estimator. In 2011, a reliability generalization meta-analysis on the Maslach Burnout Inventory (MBI) was conducted, concluding that average alpha values for the MBI dimensions ranged from 0.71 to 0.88. In the present study, we aimed to update the average reliability values of the MBI by conducting a literature search from 2010 until now and comparing to statistical procedures of meta-analysis: the Univariate approach, that were used in the previous study, and a novel meta-analytic approach based on structural equation modeling.

**Method:**

An estimation of average reliability was done based on 69 independent primary reliability coefficients for the Univariate approach. The average reliability was based on 9 independent studies in the case of the Meta-analytic Structural Equation Modeling (MASEM) approach. Given that MASEM has the additional capability of testing the internal structure of a test, we also fitted several models.

**Results:**

The data was well-suited to the bifactor model, revealing the dominance of the general factor over the domain-specific ones. Acceptable overall alpha and omega coefficients were achieved for the two of the MBI dimensions, having depersonalization reliability estimates below recommendations.

**Discussion:**

In general, the MBI can be viewed as a highly interconnected three-factor scale, being its appropriate for research purposes.

## Introduction

The Maslach Burnout Inventory (MBI) was initially published in 1981 (Maslach and Jackson, 1981). After the initial release of the original MBI, subsequent versions were progressively developed to suit various groups and settings, resulting in a current total of five versions (Maslach et al., 2018). The MBI-Human Services Survey (MBI-HSS), designed for professionals in the human services, is the original and most widely used version of the MBI (Maslach et al., 2018). The MBI-HSS was specifically adapted for medical personnel under the name of MBI-HSS (MP). The MBI-Educators Survey (MBI-ES) is a version of the original MBI for use with educators developed in 1986 (Maslach and Jackson, 1986). These three versions have 22 Likert-type items with 7 categories ranging from “never” (1) to “everyday” (7). The MBI-General Survey (MBI-GS) is a reduced version of 16 items to measure burnout in any profession (Schaufeli et al., 1996). Finally, an adaptation of the MBI-GS designed to assess burnout in college and university students was developed under the name of MBI-General Survey for Students (MBI-GS (S)). All the versions measure burnout according to the tridimensional model of burnout proposed by Maslach and Jackson (1986), that defined the burnout syndrome as an inappropriate response to chronic work stress that is characterised by emotional exhaustion (EE), depersonalization (D) and low personal accomplishment (PA).

Among all the measurement instruments that have been developed to assess the burnout syndrome, the MBI is the most widely used (Worley et al., 2008; Aguayo et al., 2011; Wheeler et al., 2011; de Beer and Bianchi, 2019). Consequently, numerous studies have been conducted to analyse their psychometric properties. Moreover, two meta-analyses were carried out to assess the average reliability of the MBI dimensions (Aguayo et al., 2011; Wheeler et al., 2011), while a separate one meta-analyzed several primary studies that assessed its internal structure (Worley et al., 2008). The results of the two reliability generalization (RG) studies concluded that both the point estimate of the average alpha coefficient and its 95% confidence intervals for emotional exhaustion and personal accomplishment dimensions were above the typically recommended cutoff point for research purposes, with a range of values between 0.87 and 0.89 for emotional exhaustion, and between 0.75 and 0.79 for personal accomplishment (Aguayo et al., 2011; Wheeler et al., 2011). However, despite that the point estimate for the depersonalization dimension was above 0.70, the 95% confidence intervals showed values between 0.68 and 0.74. This led the authors of both studies to conclude that scores on this dimension should be interpreted with caution and should not be used for making decisions such as clinical diagnoses. The results of the internal structure validity (VG) generalization study suggested that the MBI follows a model of three independent factors (Worley et al., 2008).

Although these three meta-analytic studies contributed to the evaluation of the psychometric properties of the MBI, there have several limitations. In the case of the VG meta-analysis of internal structure, only primary studies that had conducted Exploratory Factor Analysis (EFA) were included, upon which Principal Component Analysis (PCA) was performed using Varimax rotation and applying the Kaiser criterion for factor retention. This entails several drawbacks: (a) primary studies that conducted Confirmatory Factor Analysis (CFA) were not analyzed, limiting the generalization of results; (b) the models retained in EFA are often not retained when tested in CFA (Marsh et al., 2010; Morin et al., 2016); (c) the Kaiser criterion is not among the recommended procedures for factor retention, as it tends to overestimate the retained factors (Ferrando et al., 2022; Paniagua et al., 2022b); (d) Varimax rotation is not commonly used as it generates unrealistic solutions when applied in psychology studies (Lloret-Segura et al., 2014; Paniagua et al., 2022a); and (e) PCA is not strictly a type of factor analysis, as its mathematical formulation and assumptions are different. Therefore, the results of this study should be interpreted with caution, avoiding extrapolations to more common contexts in psychometrics such as those involving CFA. Furthermore, the univariate approach that the authors assumed adds some disadvantages, such as the impossibility of testing some theoretically relevant measurement models due to the scarce of primary studies that analyze these models, as well as testing which of the models perform better, differences in the sample sizes from which average effect sizes are calculated due to missing data, and, as a consequence of the former problem, differences in precision of average estimates.

In the case of the RG meta-analyses, the major limitation is that authors used alpha coefficient as the effect size taking the univariate approach, which has the following drawbacks: (a) despite alpha being the most frequently cited and commonly aggregated reliability coefficient, it is known to be based under assumptions that are unlikely to be met (Yang and Green, 2011; McNeish, 2018; Bentler, 2021), for example, the *τ*-equivalence of items or the unidimensionality of the scale, which are often violated (Gignac, 2014; Trizano-Hermosilla and Alvarado, 2016; Flora, 2020; Paniagua et al., 2022a); (b) the inclusion of more than one reliability index in the studies prevents their meta-analytic synthesis due to reporting variations; (c) the impossibility to assess the statistical dependencies among the reliability coefficients of the overall scale and the subscales; and (d) the incapability to pool the reliability index that best reflects the factor structure of the scale.

Taking into account all these drawbacks associated with univariate meta-analyses, Cheung and Chan (2005, 2009) and Cheung (2015) developed a multivariate approach to meta-analyses called Meta-Analytic Structural Equation Modeling (MASEM). Currently, various statistical procedures have been suggested for conducting MASEM that can be divided into two approaches: correlation-based and parameter-based MASEM (Jak and Cheung, 2020). The former uses correlation or covariance matrices from primary studies as effect sizes and combines them to obtain a pooled matrix that is submitted to structural equation modeling (SEM), while the latter performs SEM in each of the primary studies, and then synthesizes the resulting parameters (i.e., regression coefficients, factor loadings) as effect sizes in a meta-analysis. Within the correlation-based approach, it can be distinguished between the two-stage and one-stage MASEM techniques. The first involves two different steps: in the first place, the correlation matrices from the primary studies are synthesized using fixed- or random-effects multivariate meta-analysis (Cheung, 2015; Jak et al., 2021) and, secondly, one or more models are fitted to the pooled correlation matrix using SEM. The one-stage MASEM combines these two steps into a single one (Jak and Cheung, 2020).

Depending on the MASEM procedure used, researchers can pool the inter-item correlation matrices or the parameters (factor loadings) of a measurement model which has been fitted for each of the included studies (Cheung and Cheung, 2016). The MASEM approach has the advantage of comparing several measurement models in order to find the one that most precisely represents the test internal structure. Also, using MASEM techniques, it is possible to estimate an average and its confidence interval for the reliability index in according to all the assumptions derived from the chosen measurement model (Jak et al., 2021). However, despite its advantages, this technique has not been applied to the study of test reliability until very recently (Scherer and Teo, 2020 Blázquez-Rincón et al., 2023).

Among all the MASEM procedures, correlation-based MASEM has been considered a better one for most applications than parameter-based MASEM (Jak and Cheung, 2020; Jak et al., 2021). For example, one advantage of correlation-based over parameter-based MASEM is that it is not necessary to fit the same measurement model in all the included studies. Within the parameter-based approach, the one-stage technique is preferred over the two-stage, mainly because the latter only allows the influence of qualitative variables to be assessed through subgroup analysis, while the former allows both qualitative and continuous variables to be analyzed and their effect on the model parameters to be tested (Jak and Cheung, 2020; Jak et al., 2021). Nevertheless, they are supposed to perform equally well regarding estimation of average effect size and their confidence intervals.

Considering the limitations of the previous meta-analyses, the main goals of the present study are, on the one hand, to deepen the understanding of the psychometric properties of the MBI, and, on the other hand, to compare several procedures of psychometric meta-analysis. Specifically, we aimed (a) to apply, for the first time in the field, the correlation-based MASEM techniques to pool the inter-item Pearson’s correlation matrices of studies which have applied the MBI with the purpose, on the one hand, of testing the model that best reproduces its internal structure, and, on the other hand, of estimating its reliability; (b) to update the average reliability estimation of the MBI dimensions obtained by Aguayo et al. (2011) using the univariate approach; and (c) to compare the univariate and correlation-based MASEM approaches when estimating MBI reliability.

## Methods

The primary studies that were included in Aguayo et al. (2011) were also included in the present study. Additionally, a new literature search and synthesis was conducted in accordance with the following guidelines: Reliability Generalization Meta-Analysis (REGEMA; Sánchez-Meca et al., 2021), Preferred Reporting Items for Systematic Reviews and Meta-Analyses (PRISMA; Moher et al., 2009; Page et al., 2021), and Meta-Analysis Reporting Standards (MARS; APA Publications and Communications Board Working Group on Journal Article Reporting Standards, 2008).

### Search strategy and inclusion criteria

Three scholar databases (Web of Science, Scopus, and Central ProQuest) were examined, between January 1st of 2010 (one year before the date the previous RG meta-analyses were published) and June 1st of 2023. The following keywords were set to be found anywhere in the documents: “Maslach Burnout Inventory” and “MBI,” combined with the terms “reliability,” “accuracy,” “psychometric properties.” The flowchart presented in Supplementary Figure S1 describes the selection process of the studies. The total number of studies included in the review was 65 (with a total of 69 independent coefficient alpha estimates), resulting from combining the 45 primary studies included in Aguayo et al. (2011) with the 20 primary studies found in the current search. These 65 studies were used to perform the univariate meta-analyses. The number of studies used for the multivariate meta-analysis (MASEM approach) was considerably reduced due to the impossibility of obtaining the inter-item correlation matrices. Specifically, we used 10 independent matrices from nine studies. All the matrices were retrieved by contacting the authors through email, given that none of the studies included the inter-item correlations of the MBI in the report.

The inclusion criteria for the new identified studies were the same as in Aguayo et al. (2011): (a) to be an empirical study where the MBI-HSS or the MBI-ES was used; (b) to be based on a sample of more than one participant; and (c) to be published in peer-review journals. The following exclusion criteria were used: (a) not to be written in English, Spanish, French, Italian, and Portuguese; and (b) to be a meta-analytic or systematic review study. Besides, in the case of MASEM calculations there were two additional exclusion criteria: (a) to have administered any other MBI version that did not keep the 22-item 7-point Likert-type scale structure; and (b) to have not made available the inter-item Pearson correlation matrix.

### Data extraction

A protocol for extracting the alpha coefficients for each of the MBI dimensions along with the sample size and the inter-item Pearson correlation matrices was established. As none of the Pearson correlation matrices were available in the research report, emails were sent to all the authors of the included studies requesting them. In cases where they did not respond, two reminders were sent within a one-month period. Codification was done by two of the authors independently, yielding suitable inter-coder agreement values: For the qualitative variables, Kappa coefficients ranged from 0.95 to 1, and for the continuous variables, intraclass correlations ranging between 0.91 and 1. Inconsistencies were resolved by consensus.

### Statistical analysis

Statistical analyses were performed in R 4.1.0 (R Core Team, 2022) with the *metaSEM* (Cheung, 2015), *lavaan* (Rosseel, 2012), and *metafor* (Viechtbauer, 2010) packages.

Regarding the univariate meta-analysis, separate syntheses were carried out for the alpha coefficients (Cronbach, 1951) reported for each of the MBI dimensions. Using the transformation proposed by Bonett (2002), alpha coefficients were transformed to normalize their distributions and stabilize their variances following the recommendations by Sánchez-Meca et al. (2013).

As fixed- and random-effects models were used, the alpha coefficients were weighted by the inverse variance method, where, in the case of former, the variance is the within-study variance, and, in the case of the latter, the variance is the sum of the within-study and the between-studies variances. Between-study variance, *τ*^2^, was estimated using the Paule and Mandel estimator (Boedeker and Henson, 2020). The 95% confidence interval around each overall reliability estimates were computed with the method proposed by Hartung (1999). The degree of heterogeneity was assessed with the *Q* test and the *I*^2^ index, with values of approximately 25, 50, and 75% representing low, moderate, and large heterogeneity (Higgins et al., 2003). Predictions intervals around overall reliability estimates were also computed for heterogeneity assessment (Borenstein et al., 2021).

The statistical analyses for MASEM were based on Scherer and Teo (2020) tutorial. The employed effect size was the Pearson correlation between pair of items, resulting in a combined inter-item correlation matrix. This can be done using the fixed-effect model, which assumes that bivariate correlations vary only due to sampling error, or using the random-effects model, which is based on the assumption that there are relevant (not only due to sampling error) heterogeneity among the correlations (Sánchez-Meca et al., 2013; Borenstein et al., 2021).

Concurrently (in the case of the one-stage MASEM) or subsequently (in the case of the two-stage MASEM), the measurement model is submitted to structural equation modeling employing the combined inter-item correlation matrix. We fitted five measurement models employing weighted least square as the method for parameters estimation: (a) a one-factor congeneric model, with freely estimated factor loadings; (b) a three-factor model with correlated factors, which is the proposed originally by Maslach and Jackson (1981); and (c) a bifactor model with one general factor and three domain-specific factors (EE, D, and PA); (d) the one-factor and (e) the three-factor models *τ*-equivalent versions were also fitted, given that alpha coefficient is mostly appropriate for *τ*-equivalent models and that it is the most widely reported reliability estimate in univariate meta-analysis.

In real scenarios, discovering factor structures which are, on the one hand, entirely unidimensional or, on the other hand, multidimensional with zero covariances between factors is improbable (Ondé and Alvarado, 2022). Hence, the bifactor model was also included because it is a suitable psychometric tool for testing the essential unidimensionality of a test; that is, a factor structure that is not strictly unidimensional and for which multidimensional models yield a better account of the correlations among the items (Reise, 2012; Brown, 2015). More precisely, bifactor models account for the item variability dividing it into two sources: the one that is explained by a general factor, and the one that is explained by the specific-domain factors. When omega hierarchical values are above 0.70, it is recommended to assume essential unidimensionality (Reise et al., 2013; Rodriguez et al., 2016a,b).

The measurement models were assessed with the χ2 statistic and the global fit indices CFI, TLI, RMSEA, and SRMR (Brown, 2015; Kline, 2015). TLI and CFI values above 0.95 are considered adequate, while SRMR values below 0.08 are acceptable (Hu and Bentler, 1999). RMSEA values below 0.06 are reasonable (Hu and Bentler, 1999) and below 0.05 are seen as evidence of a satisfactory fit (Browne and Cudeck, 1993). Likelihood-ratio tests and AIC were applied for all the models that yielded adequate fit indices.

The reliability index was selected in accordance with and after determining the measurement model that most accurately reflect the internal structure of the MBI. All the formulas for calculating each of the selected reliability indices can be consulted in Supplementary Table S1. Coefficient alpha was considered for the τ -equivalent models, whereas omega total coefficient was considered for the congeneric measurement models. Omega hierarchical was the reliability index that accounted for the variance of the general factor when a bifactor model was fitted, whereas omega subscale was used to account for the variance of the domain-specific factors. The point estimates for the subscales reliability were calculated from the estimated factor loadings obtained for the fitted measurement model, and its standard errors were computed applying the delta method (Raykov and Marcoulides, 2004). These standard errors were used to calculate a confidence interval around the point estimates ( α=0.05 ). As the consequence of the fact that reliability coefficients are truncated in the interval [0, 1] their distributions are skew. This is the reason why it is proper to obtain a confidence interval from a monotonic increasing transformation of the reliability coefficient, such as the logit function (see Equation 1), that makes it unbounded (Browne, 1982):


(1) $$k=\ln\left(\frac{\hat{\rho }}{1-\hat{\rho }}\right)$$

with $\ln\left(\text{.}\right)$ denoting the natural logarithm and $\hat{\rho }$ denoting the point estimate for the scale reliability. The standard error associated with $\hat{k}$ can also be furnished via the delta method (see Equation 2) (Browne, 1982):


(2) $${SE}_{k}=\frac{{SE}_{\hat{\rho }}}{\hat{\rho }\left(1-\hat{\rho }\right)}$$

where ${SE}_{\hat{\rho }}$ denoted the standard error for the scale reliability. Once the point estimate and variability of the scale reliability have been transformed, a Wald-type confidence interval can be obtained by computing the lower and upper limits as $k_{\mathrm{lower}}=k-Z_{1-\alpha /2}\left({SE}_{k}\right)$ and $k_{\mathrm{upper}}=k+Z_{1-\alpha /2}\left({SE}_{k}\right)$ , respectively, where $Z_{1-\alpha /2}$ is the $100\left(1-\alpha /2\right)$ quantile of the standard normal distribution (see Equation 3). In the last place, $k_{\mathrm{lower}}$ and $k_{\mathrm{upper}}$ were back transformed to obtain a $100\left(1-\alpha /2\right)$ % confidence interval for the scale reliability by using the logistic function or inverse of the logit function:


(3) $$\left[1/\left(1+e^{-k_{\mathrm{lower}}}\right),\;1/\left(1+e^{-k_{\mathrm{upper}}}\right)\right]$$

## Results

### Descriptive characteristics of the studies

References of new included studies are available at the Supplementary material. The total sample was 38,797 subjects, most of whom (25.93%) were men. The distribution of sample sizes was highly skewed and leptokurtic, with a mean of 546.4 subjects per sample (median = 319, SD = 783.02, skewness = 5.27, kurtosis = 34.17).

### Univariate reliability generalization

We collected 65 primary studies that reported the alpha coefficient for each of the MBI dimensions, yielding 69 independent estimates with 37,160 participants. Table 1 shows the pooled alpha coefficients, under both fixed- and random-effects models, for each of the MBI dimensions and their respective confidence limits once back transformed to alpha coefficients in order to facilitate the interpretation. For all dimensions, the mean coefficients were above 0.70. Evidence of heterogeneity was found for all the pooled coefficients, with all *Q* statistics being significant (*p* < 0.001) and *I*^2^ indexes ranging between 94 and 95%.

**Table 1:** Average alpha coefficients and heterogeneity results for the MBI (*k* = 69).

Dimension	${\bar{\alpha }}_{FE}$	95% CI	${\bar{\alpha }}_{RE}$	95% CI	*Q*	*I^2^*	PI
EE	0.876	[0.869, 0.883]	0.876	[0.866, 0.884]	847.3***	94.9	[0.777, 0.931]
D	0.685	[0.662, 0.706]	0.706	[0.684, 0.736]	1308.4***	94.5	[0.485, 0.832]
PA	0.768	[0.752, 0.784]	0.770	[0.725, 0.787]	1251.9***	95.2	[0.582, 0.873]

*k*, independent samples; EE, emotional exhaustion; D, depersonalization; PA, personal accomplishment; ${\bar{\alpha }}_{FE}$ , average alpha coefficient under the fixed-effect model; ${\bar{\alpha }}_{RE}$ , average alpha coefficient under the random-effect model; CI, confidence interval; *Q*, Cochran’s heterogeneity *Q* statistic with *k* − 1 degrees of freedom; *I*^2^, heterogeneity index; PI, prediction interval. ***p < 0.001.

### Meta-analytic structural equation modeling

#### Meta-analytic synthesis and measurement model assessment

**Figure 1:**
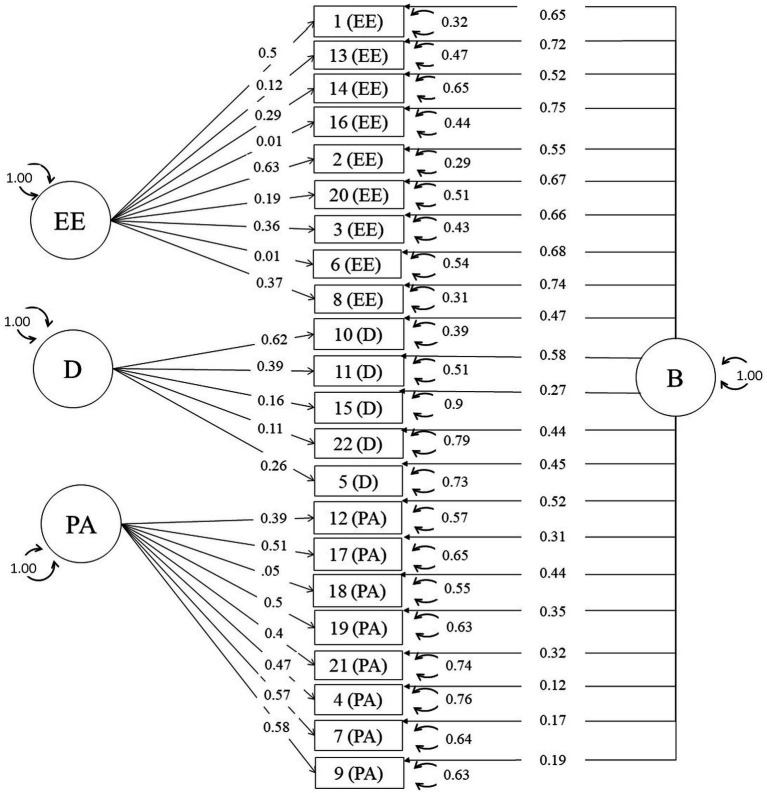
Standardized solution for the bifactor model with two-stage MASEM.

**Figure 2:**
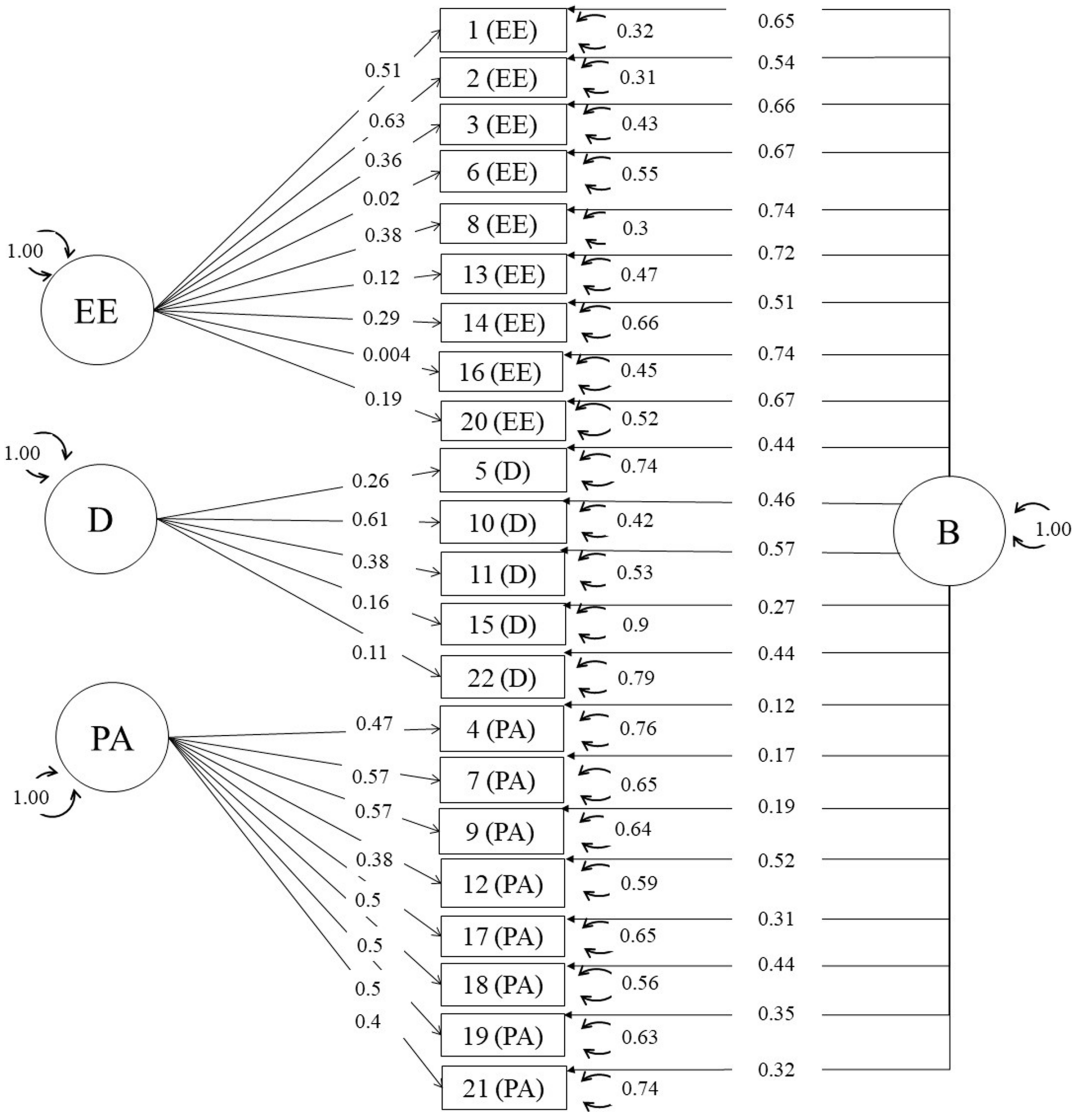
Standardized solution for the bifactor model with one-stage MASEM.

Among the 10 matrices included, the one from Lin et al. (2022) was nonpositive definite, being consequently excluded from the subsequent analyses. In two-stage MASEM, there are two steps. Firstly, the pooled correlation matrix is estimated, and subsequently the measurement model is fitted via SEM. Given the small number of studies, we decided to assume a fixed-effect meta-analytic model. Supplementary Table S2 shows the estimated pooled correlation inter-item matrix. In one-stage MASEM, these two steps are done all together. Figures 1, 2 depicts the estimates for the parameters of the bifactor model for the one- and two-stage MASEM procedures, while the factor structure of the rest of the models can be consulted in Supplementary Figures S2–S9.

Fit indices of all the tested models can be seen in Tables 2, 3. For the two MASEM procedures, RMSEA values were adequate for the three-factor congeneric and the bifactor models, whereas SRMR, CFI and TLI values were far from the recommended cut-off points for the all the models except for bifactor. CFI and TLI indices for the one-stage MASEM could not be computed due to problems fitting the independence model.

**Table 2:** Fit indices for the measurement models with two-stage MASEM (*k* = 9).

Measurement model (fixed-effects correlations)	$\chi^{2}\left(df\right)$	CFI	TLI	RMSEA [95% CI]	SRMR
One-factor τ -equivalent	5928.04 (230)*	0.584	0.582	0.088 [0.086, 0.090]	0.194
One-factor congeneric	3189.10 (209)*	0.782	0.759	0.067 [0.065, 0.069]	0.169
Three-factor τ -equivalent	3233.12 (225)*	0.780	0.774	0.065 [0.063, 0.067]	0.110
Three-factor congeneric	2031.72 (206)*	0.867	0.851	0.053 [0.051, 0.055]	0.099
Bifactor	1173.58 (187)*	0.928	0.911	0.041 [0.039, 0.043]	0.058

*k*, inter-item correlation matrices; χ2 , chi-squared statistic; df, degrees of freedom. * = *p* < 0.001.

**Table 3:** Fit indices for the measurement models with one-stage MASEM (*k* = 9).

Measurement model (fixed-effects correlations)	$\chi^{2}\left(df\right)$	CFI	TLI	RMSEA [95% CI]	SRMR
One-factor τ -equivalent	5793.02 (230)*	—	—	0.087 [0.085, 0.089]	0.165
One-factor congeneric	3110.03 (209)*	—	—	0.066 [0.064, 0.068]	0.164
Three-factor τ -equivalent	2157.15 (225)*	—	—	0.064 [0.062, 0.066]	0.107
Three-factor congeneric	1978.95 (206)*	—	—	0.052 [0.049, 0.054]	0.097
Bifactor	1151.82 (187)*	—	—	0.040 [0.038, 0.043]	0.057

*k*, inter-item correlation matrices; χ2 , chi-squared statistic; df, degrees of freedom. * = *p* < 0.001.

The bifactor model showed a satisfactory fit to the data, yielding, in the case of the two-stage MASEM, an omega hierarchical equal to 0.89 for the total burnout scale, an omega subscale values equal to 0.12 for the EE dimension, 0.20 for the D dimension, and 0.63 for the PA dimension (see Table 4). For the one-stage MASEM, omega hierarchical coefficient was 0.87 for the total burnout scale, and omega subscale coefficients were 0.44 for the EE dimension, 0.35 for the D dimension, and 0.64 for the PA dimension (see Table 5). As suggested by Ondé and Alvarado (2022), we employed the bifactor model as a psychometric tool for evaluating the essential unidimensionality of the MBI. These results suggests that the MBI is an essentially unidimensional scale, in which prevails a general factor over the three specific domains (EE, D, and PA).

**Table 4:** Average reliability coefficients with two-stage MASEM.

Measurement model	${ES}_{B}^{+}$ [95% CI]	${ES}_{EE}^{+}$ [95% CI]	${ES}_{D}^{+}$ [95% CI]	${ES}_{PA}^{+}$ [95% CI]
One-factor τ -equivalent	0.869 [0.865, 0.873]	—	—	—
One-factor congeneric	0.930 [0.926, 0.931]	—	—	—
Three-factor τ -equivalent	—	0.869 [0.865, 0.873]	0.642 [0.640, 0.650]	0.768 [0.766, 0.712]
Three-factor congeneric	—	0.915 [0.911, 0.919]	0.698 [0.682, 0.713]	0.807 [0.797, 0.816]
Bifactor	0.892 [0.888, 0.897]	0.124 [0.093, 0.158]	0.204 [0.172, 0.235]	0.628 [0.604, 0.650]

B, burnout; EE, emotional exhaustion; D, depersonalization; PA, personal accomplishment; ${ES}^{+}$ , average effect size (omega total in the congeneric models, omega hierarchical, for the burnout total scale, and omega subscale, for the MBI dimensions, in the bifactor model, and alpha in the τ -equivalent models); CI, confidence interval.

**Table 5:** Average reliability coefficients with one-stage MASEM.

Measurement model	${ES}_{B}^{+}$ [95% CI]	${ES}_{EE}^{+}$ [95% CI]	${ES}_{D}^{+}$ [95% CI]	${ES}_{PA}^{+}$ [95% CI]
One-factor τ -equivalent	0.869 [0.865, 0.873]	—	—	—
One-factor congeneric	0.928 [0.925, 0.931]	—	—	—
Three-factor τ -equivalent	—	0.869 [0.865, 0.873]	0.642 [0.640, 0.650]	0.768 [0.766, 0.712]
Three-factor congeneric	—	0.912 [0.908, 0.916]	0.689 [0.673, 0.704]	0.804 [0.794, 0.813]
Bifactor	0.872 [0.865, 0.879]	0.443 [0.377, 0.511]	0.346 [0.307, 0.387]	0.640 [0.608, 0.680]

B, burnout; EE, emotional exhaustion; D, depersonalization; PA, personal accomplishment; ${ES}^{+}$ , average effect size (omega total in the congeneric models, omega hierarchical, for the burnout total scale, and omega subscale, for the MBI dimensions, in the bifactor model, and alpha in the τ -equivalent models); CI, confidence interval.

#### Average reliability

Tables 4, 5 show the estimated reliability coefficients for the corresponding factors regarding each measurement model. Alpha coefficient was calculated for *τ*-equivalent models, while omega total was the reliability index for congeneric models. Omega hierarchical (in the case of burnout total score) and omega subscale (in the case of MBI dimensions) was reported for the bifactor model. Results were very similar with both MASEM procedures. Except for depersonalization in the three-factor congeneric model, the point estimate of average reliability, and its 95% confidence intervals, of the burnout total score and burnout dimensions in all models were from adequate to excellent (from 0.72 to 0.93).

Given that, according to our results, a total burnout score can be reported, a reliability index suitable for this essential unidimensionality, like omega total, might be computed. In the present work, using the two-stage MASEM, omega total for the whole scale reached 0.867 [95% CI: (0.874, 0.880)] in the bifactor model, and using one-stage MASEM, it reached 0.892 (95% CI: [0.887, 0.897]).

## Discussion

The goal of the current work was comparing several approaches of psychometric meta-analysis taking the Maslach Burnout Inventory (MBI) as measurement instrument by (a) updating the average reliability estimation of the MBI dimensions obtained by Aguayo et al. (2011) with the univariate approach; (b) applying the MASEM approach to combine the inter-item Pearson correlation matrices obtained from studies that have applied the MBI in order to test the measurement model that best reflect its internal structure and to estimate the reliability of the MBI dimensions; and (c) comparing the results of univariate and MASEM approaches.

The MBI measures the burnout syndrome according the tridimensional theory proposed by Maslach and Jackson (1981), that defined the burnout syndrome as an inappropriate response to chronic work stress that is characterised by emotional exhaustion (EE), depersonalization (D) and low personal accomplishment (PA). The psychometric properties of the MBI have been analyzed by numerous studies, including one meta-analysis that synthesized coefficients of its internal structure (Worley et al., 2008), and another two that averaged reliability coefficients of its three dimensions (Aguayo et al., 2011; Wheeler et al., 2011).

Although all these meta-analytic studies (and the rest of empirical studies) are valuable to understand the psychometric properties of the MBI, more analyses should be done because of two main reasons: First, the limitations that these studies had, and second, there have been proposed new statistical procedures to perform meta-analyses that try to overcome some drawbacks of the standard approach. Regarding the meta-analysis on internal structure validity (also known as Validity Generalization studies), some important limitations refer to the fact that only primary studies that performed Exploratory Factor Analysis (EFA) were synthesized and the use of a combination of statistical procedures that are not recommended currently, such as Principal Component Analysis, Varimax rotation, and the Kaiser rule (Lloret-Segura et al., 2014; Ferrando et al., 2022; Paniagua et al., 2022a).

Regarding the meta-analyses of reliability coefficients (also known as Reliability Generalization studies), the main drawback, that is also shared with the Validity Generalization meta-analysis by Worley et al. (2008), concerns the use of the univariate approach to meta-analysis, that implies several disadvantages: (a) while alpha is commonly cited and is the predominant combined reliability coefficient, it is widely recognized to rely on assumptions that are not likely to be met (Yang and Green, 2011; McNeish, 2018; Bentler, 2021), for example, the *τ*-equivalence of items or the unidimensionality of the scale, which are often violated (Gignac, 2014; Trizano-Hermosilla and Alvarado, 2016; Flora, 2020; Paniagua et al., 2022a); (b) the diverse of reliability indices reported in the included studies hinders the possibility of synthesizing their results; (c) the fact that the interdependencies among the reliability coefficients of general scale and subscales cannot be examined; and (d) the incapability to pool the reliability coefficient that accurately reflects the factor structure of the scale.

Given the limitations mentioned above, a new statistical analysis technique denominated Meta-Analytic Structural Equation Modeling (MASEM) was employed to overcome these issues. Although this technique was originally proposed by Cheung and Chan (2005, 2009) almost a decade ago (Cheung, 2015), it has only been recently applied to the study of test reliability (Scherer and Teo, 2020; Blázquez-Rincón et al., 2023).

### Internal structure and reliability generalization

In this study, it was observed that neither the *τ*-equivalent nor the congeneric one- and three-factor models adequately fitted the combined inter-item correlation matrix. However, the bifactor model demonstrated satisfactory fit indices.

Following several recommendations (Reise, 2012; Rodriguez et al., 2016b), the bifactor model was employed to evaluate the essential unidimensionality of the MBI. That is, assessing the proportion of item variability attributed to specific factors (emotional exhaustion, depersonalization, and personal accomplishment) after accounting for a general (burnout) factor. The average omega hierarchical coefficient, with a value of 0.89, exceeded the recommended cut-off of 0.70, which suggests essential unidimensionality (Reise et al., 2013; Rodriguez et al., 2016a,b). However, average omega subscale coefficients for the three dimensions were below this threshold. Hence, once the general factor was incorporated, the specific factors accounted for only a small portion of the true score variance. As the MBI can be viewed as an essentially unidimensional scale, it could be deemed appropriate to compute a total burnout score and report reliability using omega total.

These results are in line with the findings of Aguayo-Estremera et al. (2023), which showed that whereas the three-factor congeneric model did not reach adequate global fit indices, the bifactor model fitted the data well. Despite these results, Aguayo-Estremera et al. (2023) did not advocate for a unidimensional factor structure of the MBI because of two reasons: In the first place, a good fit for the bifactor model does not imply evidence for unidimensional structures; in the second place, they fitted a three-factor model using Exploratory Structural Equation Modeling (ESEM) that obtained excellent global fit indices. The difference between CFA and ESEM is that in the latter cross-loadings are specified in the model, allowing a better model fit in the case of relevant cross-loadings. Hence, base in the current study results, we cannot claim that the MBI is not best represented by a three-dimensional structure, since ESEM analysis may show evidence for it.

In contrast to the guidance provided by the American Psychological Association (Appelbaum et al., 2018) who argued that researchers ought to disclose a reliability index suitable for the characteristics of the test, out of the 65 studies included, 89.2% reported the alpha coefficient without considering the assumptions associated with the measurement model (Green and Yang, 2009). Just 10.8% (seven studies) reported some omega coefficient, which is a more suitable reliability index (Trizano-Hermosilla and Alvarado, 2016) given that neither the one-nor the three-factor *τ*-equivalent models fitted the data adequately. In the current study the average omega total of the whole inventory (ranging from 0.87 to 0.89) was higher than that of the three subscales (ranging from 0.12 to 0.64).

### Comparisons between the univariate and MASEM approaches

The univariate approach is largely the most used statistical technique to perform meta-analytic studies, mainly because the novelty of the MASEM approach, that was originally proposed by Cheung and Chan (2005). According to Scherer and Teo (2020), the univariate approach applied to meta-analysis of reliability coefficients has several disadvantages, such as, the loss of studies that do not report any reliability index or because there are different coefficients that cannot be combined, the fact that statistical dependencies among the reliability coefficients of the general scale and the subscales cannot be assessed, the incapability to pool the reliability index that most accurately reflects the factor structure of the inventory, and the incapacity to test a *τ*-equivalent model for which alpha coefficient is appropriate.

Some of these limitations have been corroborated in the present study, for example, the inability to test the fit of several measurement models, including a model that is appropriate for alpha coefficient. With the univariate approach, only alpha reliability index could be calculated, assuming that the three-factor *τ*-equivalent model fitted the data well, which was not the case as observed using the MASEM approach. Likewise, reliability index for any other theoretically relevant measurement model (e.g., bifactor) could not be estimated, since few of the primary studies (four, in the case of the bifactor model) tested other models.

However, with the univariate approach to synthesizing alpha coefficients the number of studies included was higher than with the MASEM approach. Even though the latter approach has more statistical capacity to deal with missing data than the former, the necessary information to perform the analyses, which is the Pearson inter-item correlation matrix, was usually not reported (and difficult to recover contacting to authors). Conversely, alpha coefficient was frequently reported, leading to include in the synthesis an elevate number of primary effect sizes.

Regarding average reliability coefficients, several results are worth discussing. Firstly, it is admissible to compare the results of the three-factor *τ*-equivalent model with univariate and MASEM approaches, given that alpha coefficient is the proper reliability index to be used. Under the fixed-effect model, the results showed very similar values for all the procedures, except for depersonalization dimension for which average alpha value was higher in the univariate approach.

Secondly, we can also compare the results of the congeneric models within the MASEM approach. The results were similar in both statistical procedures for the one- and three-factor congeneric models, but not for bifactor, in which omega coefficients were higher for the one-stage MASEM than for the two-stage MASEM. Hence, we can conclude that, as expected, both MASEM techniques lead to similar results and broadly the same interpretations.

Thirdly, it is interesting to assess the differences in reliability indices as a function of the model type and fit to the data. For the one- and the three-factor models, it was observed that alpha were lower than omega values. This discrepancy might be because *τ*-equivalent models did not fit the data well, leading to bias (i.e., underestimation) in the reliability estimators. This result is especially important for depersonalization dimension, which showed alpha values below 0.70. This might also explain variations in primary studies results, which inconsistently fall above and below this threshold, leading some authors to claim that depersonalization scores should be interpreted with caution (Aguayo et al., 2011; Wheeler et al., 2011).

Regarding factor loadings withing the MASEM approach, both statistical techniques performed almost equally for *X*^2^, RMSEA and SRMR indices. The biggest difference was that CFI and TLI indices could not be computed using one-stage MASEM because of a failure fitting the independence model. As with reliability indices, it seems that both MASEM techniques yield analogous conclusions.

### Limitations and future studies

Some of the limitations of the current study come from the characteristics of the statistical procedures themselves. Firstly, we excluded from the MASEM analyses one study (Lin et al., 2022) meeting the selection criteria analyses because the matrix was nonpositive definite and these matrices cannot be synthesized. Additionally, several authors did not respond when contacted to request the correlation matrices.

Secondly, two studies (de Beer and Bianchi, 2019; Schneider et al., 2020) that also meet the selection criteria could not be included in in the univariate analyses because the full text was not available in the bibliographic databases of our academic institutions. Therefore, we urge authors of primary studies to preregister and report their data in a repository, like PsyArXiv or Open Science Framework.

Thirdly, we could not compare the results of two global fit indices (CFI and TLI) between the two MASEM techniques since there was a computational problem in fitting the independence models with the one-stage procedure. This procedure is highly complex and time demanding, so it might be common to came across this issue. Future studies should explore this topic in order to optimize the procedure.

Fourthly, we could not fit three-factor model using ESEM within MASEM approach as this procedure is yet not developed. Given that other studies have obtained good results for the three-factor model in which cross-loadings are modeled (as can be done with ESEM), it would be very useful to expand the MASEM approach so that ESEM analysis was allowed.

Finally, our results are restricted to the estimation of average effect sizes and their confidence intervals. We did not include a comparison regarding moderator analyses, so it would be desirable for future studies to delve into this line of research.

## Conclusion

This study represents the inaugural use of an innovative meta-analytic technique, grounded in structural equation modeling, to scrutinize the psychometric properties of the widely employed Maslach Burnout Inventory (MBI). We have made a meaningful contribution to the examination of the internal structure of such a controversial issue, concluding that the MBI can be viewed as an essentially unidimensional scale, for which it is admissible to estimate a total burnout factor score. The application of a bifactor model reveals that a single burnout factor takes primacy over the three specific factors (emotional exhaustion, depersonalization, and personal accomplishment) in explaining item variance. However, this conclusion does not necessarily lead to rejecting a three-dimensional structure as proposed by the original authors (Maslach and Jackson, 1981). As obtained previously (Aguayo-Estremera et al., 2023), evidence for a three-factor model might be obtained when controlling for cross-loadings using Exploratory Structural Equation Modeling (ESEM). Likewise, we can state that the one-factor model is not a proper representation of the MBI internal structure since fit indices for this model were poor.

Considering the congeneric models, the average reliability values showed by the one- and three-factor models were good (from 0.77 to 0.93), except for depersonalization dimension, which can be below 0.70. Due to the poor fit of the *τ*-equivalent models to the data and the common reporting of MBI scores for its three dimensions, we consider that computing an omega total coefficient for these three dimensions serves as a more appropriate reliability measure for the MBI compared to other coefficients derived from a one-factor model. Similarly, our findings also endorse the utilization of a burnout total score that can be used in applied and research settings.

## Data availability statement

The original contributions presented in the study are included in the article/Supplementary material, further inquiries can be directed to the corresponding author.

## Author contributions

RA-E: Visualization, Conceptualization, Formal analysis, Investigation, Writing – original draft. GC-DF: Methodology, Resources, Software, Writing – review & editing, Funding acquisition. TA: Methodology, Writing – review & editing, Investigation, Validation, Visualization. EO-C: Methodology, Visualization, Writing – review & editing, Data curation, Software. JG-U: Methodology, Software, Writing – review & editing, Formal analysis, Investigation, Resources, Validation. JR-B: Resources, Software, Validation, Visualization, Writing – review & editing. EF-S: Conceptualization, Investigation, Funding acquisition, Project administration, Supervision, Visualization, Writing – review & editing.
