# Supplementary material for: A comparison of univariate and meta-analytic structural equation modeling approaches to reliability generalization applied to the Maslach Burnout Inventory
Source: Front Psychol. 2024 May 3;15:1383619. doi: 10.3389/fpsyg.2024.1383619 (PMC11110656; doi:10.3389/fpsyg.2024.1383619)
Supplement: Supplementary file 1 [file Data_Sheet_1.PDF]

A comparison of Univariate and Meta-Analytic Structural Equation Modeling Approaches to Reliability Generalization applied to the Maslach Burnout Inventory

Supplemental material

Table S1

Formulas for the reliability indices

Reliability coefficient	*Formula*	*Reference*
Alpha	$\frac{J}{J-1}\left[1-\frac{\sum_{1\leq i\neq k\leq J}^{J}(\sigma_{ik})}{\sigma_X^2}\right]$	Cronbach (1951)
Omega total	$\frac{\left(\sum_{i=1}^{J}\lambda_i\right)^2}{\left(\sum_{i=1}^{J}\lambda_i\right)^2+\sum_{i=1}^{J}\sigma_{\varepsilon\,i}^2}$	McDonald (1999)
Omega hierarchical	$\frac{\left(\sum_{i=1}^{J}\lambda_i^{(g)}\right)^2}{\left(\sum_{i=1}^{J}\lambda_i^{(g)}\right)^2+\sum_{i=1}^{J}\sigma_{\varepsilon\,i}^2+\left(\sum_{i=1}^{J_1}\lambda_i^{(s_1)}\right)^2+\cdots+\left(\sum_{i=1}^{J_p}\lambda_i^{(s_p)}\right)^2}$	McDonald (1999)
Omega subscale	$\frac{\left(\sum_{i=1}^{J_h}\lambda_i^{(s_h)}\right)^2}{\left(\sum_{i=1}^{J_h}\lambda_i^{(g)}\right)^2+\sum_{i=1}^{J_h}\sigma_{\varepsilon\,i}^2+\left(\sum_{i=1}^{J_h}\lambda_i^{(s_h)}\right)^2}$	Reise, Bonifay, & Haviland (2013)

Note. Formulas for the reliability indices computed depending on the measurement model fitted to the included studies. J = number of items; σ_{ik}= covariance between item *i* and item *k*; σ_X^2 = test variance; λ_i = factor loading for item *i*; $\sigma_{\varepsilon i}^2$ = error variance for item *i*; $\lambda_i^{(g)}$= factor loading of item *i* on the general factor *g*; $\lambda_i^{(s_1)}$ ..., $\lambda_i^{(s_h)}$, ... $\lambda_i^{(s_p)}$= factor loadings of item *i* on the specific factors s_1, ..., s_h, ..., s_p; and the specific factors comprise J_1, ..., J_h, ..., J_p items. Formula for Alpha was adapted from "Coefficient alpha and the internal structure of tests," by L. J. Cronbach, 1951, Psychometrika, 16(3), p.297 (https://doi.org/10.1007/BF02310555). Copyright 1951 by the Psychometric Society. Omega total and Hierarchical formulas were adapted from Test theory: A unified treatment (p. 119), by R. P. McDonald, 1999 (https://doi.org/10.4324/9781410601087). Copyright 1999 by Taylor & Francis Group. Omega subscale formula was adapted from "Scoring and modeling psychological measures in the presence of multidimensionality", by S. P. Reise, W. E. Bonifay, & M. G. Haviland, 2013, Journal of Personality Assessment, 95(2), p.129 (https://doi.org/10.1080/00223891.2012.725437) Copyright 2013 by Taylor & Francis Group, LLC.

Table S2

Estimated Pooled Inter-Item Correlation Matrix Under Fixed-Effect Model

	1	2	3	4	5	6	7	8	9	10	11	12	13	14	15	16	17	18	19	20	21	22
1 (EE)	1																					
2 (EE)	.66	1																				
3 (EE)	.56	.56	1																			
4 (PA)	.05	.06	.05	1																		
5 (DE)	.23	.18	.24	.14	1																	
6 (EE)	.38	.35	.36	.03	.24	1																
7 (PA)	.07	.05	.06	.36	.15	.08	1															
8 (EE)	.64	.58	.58	.04	.25	.42	.07	1														
9 (PA)	.09	.07	.08	.29	.11	.06	.39	.12	1													
10 (DE)	.25	.21	.25	.12	.33	.25	.09	.29	.07	1												
11 (DE)	.33	.26	.32	.08	.28	.32	.08	.38	.1	.5	1											
12 (PA)	.32	.26	.38	.19	.2	.24	.25	.35	.28	.2	.22	1										
13 (EE)	.46	.38	.43	.06	.28	.36	.09	.55	.14	.33	.39	.32	1									
14 (EE)	.42	.43	.35	.06	.16	.3	.05	.45	.06	.2	.27	.15	.4	1								
15 (DE)	.08	.08	.1	.13	.26	.15	.14	.12	.09	.2	.17	.1	.17	.09	1							
16 (EE)	.42	.36	.41	.06	.21	.53	.08	.45	.1	.28	.37	.26	.42	.37	.15	1						
17 (PA)	.14	.11	.18	.27	.2	.11	.31	.18	.3	.13	.12	.3	.21	.08	.11	.16	1					
18 (PA)	.23	.19	.23	.2	.19	.19	.23	.28	.31	.16	.2	.41	.26	.12	.09	.25	.39	1				
19 (PA)	.13	.08	.19	.18	.16	.17	.26	.18	.33	.17	.16	.32	.23	.11	.09	.19	.32	.43	1			
20 (EE)	.48	.42	.48	.05	.2	.32	.09	.55	.11	.29	.36	.35	.47	.33	.14	.39	.15	.23	.17	1		
21 (PA)	.19	.14	.19	.19	.14	.14	.25	.18	.23	.13	.13	.31	.18	.12	.11	.21	.3	.3	.29	.17	1	
22 (DE)	.25	.19	.2	.07	.26	.23	.1	.24	.08	.24	.25	.15	.3	.21	.17	.28	.16	.15	.12	.25	.11	1

Note. EE = Emotional Exhaustion; DE = Depersonalization; PA = Personal Accomplishment.

Figure S1

Flowchart of the selection process.

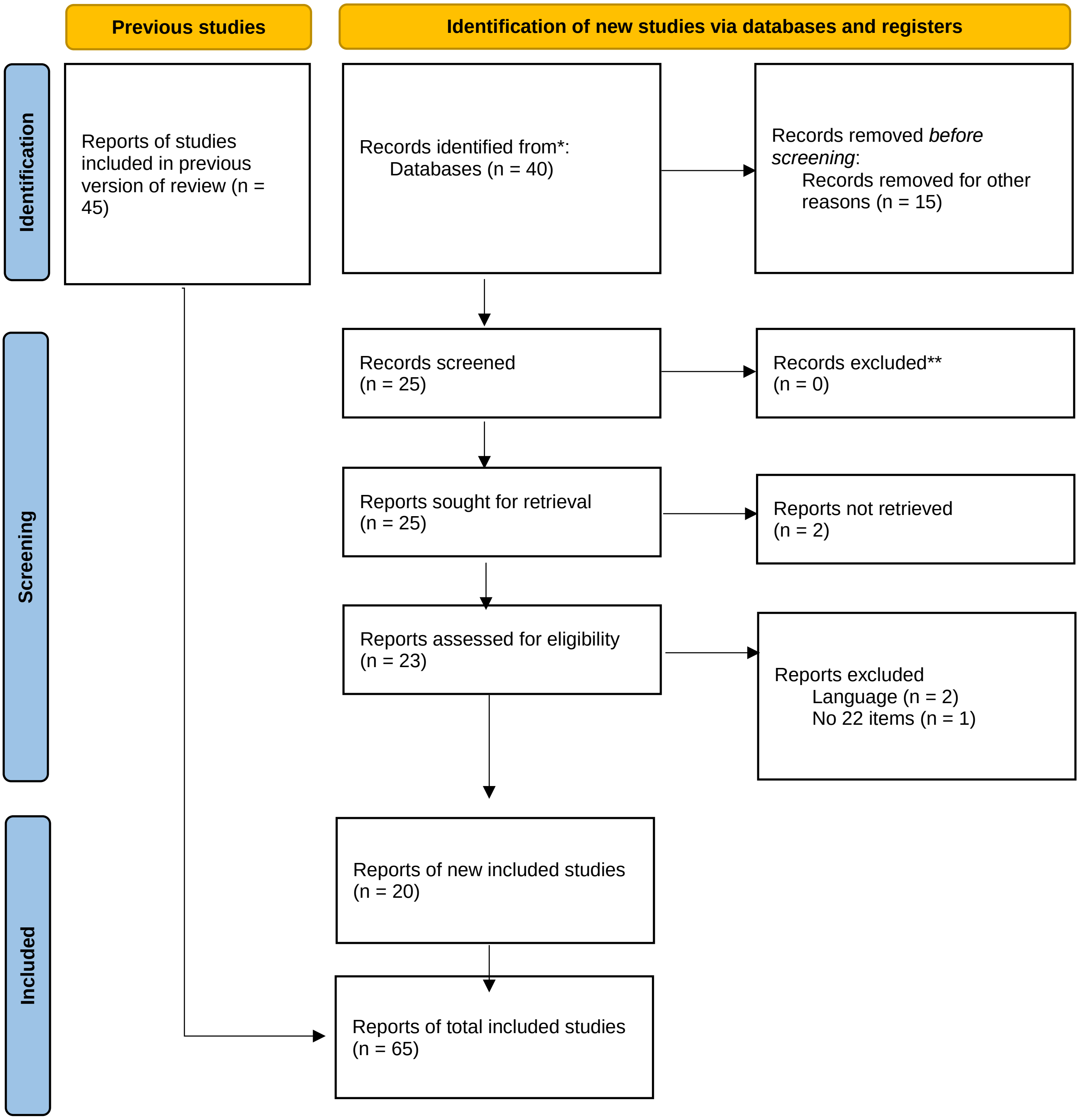

Figure S2

Solution for the one-factor τ-equivalent model with two-stage MASEM

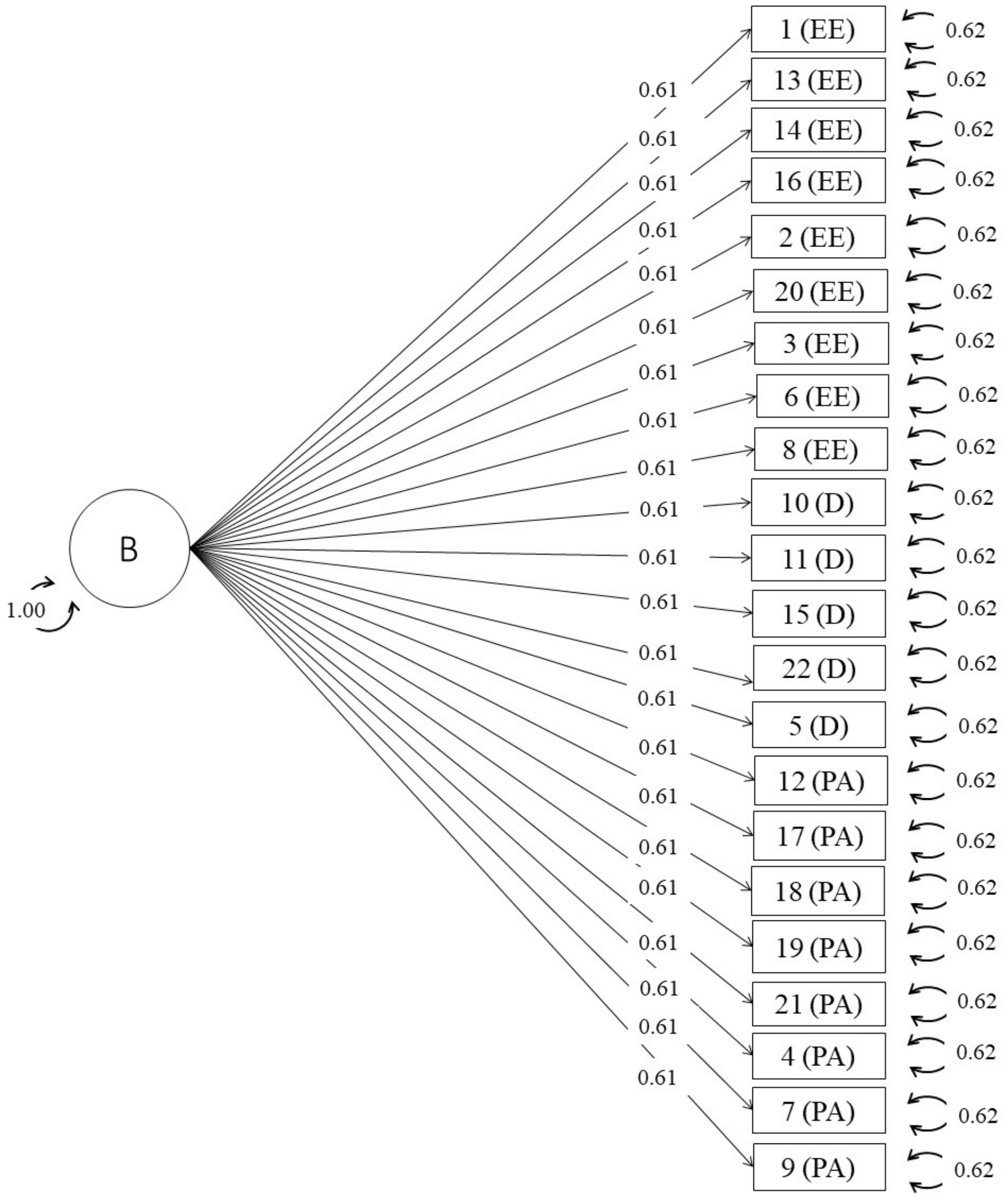

Note. Standardized solution for the bifactor model fitted to the 9 inter-item correlation matrices of the included studies.

Figure S3

Solution for the one-factor congeneric model with two-stage MASEM

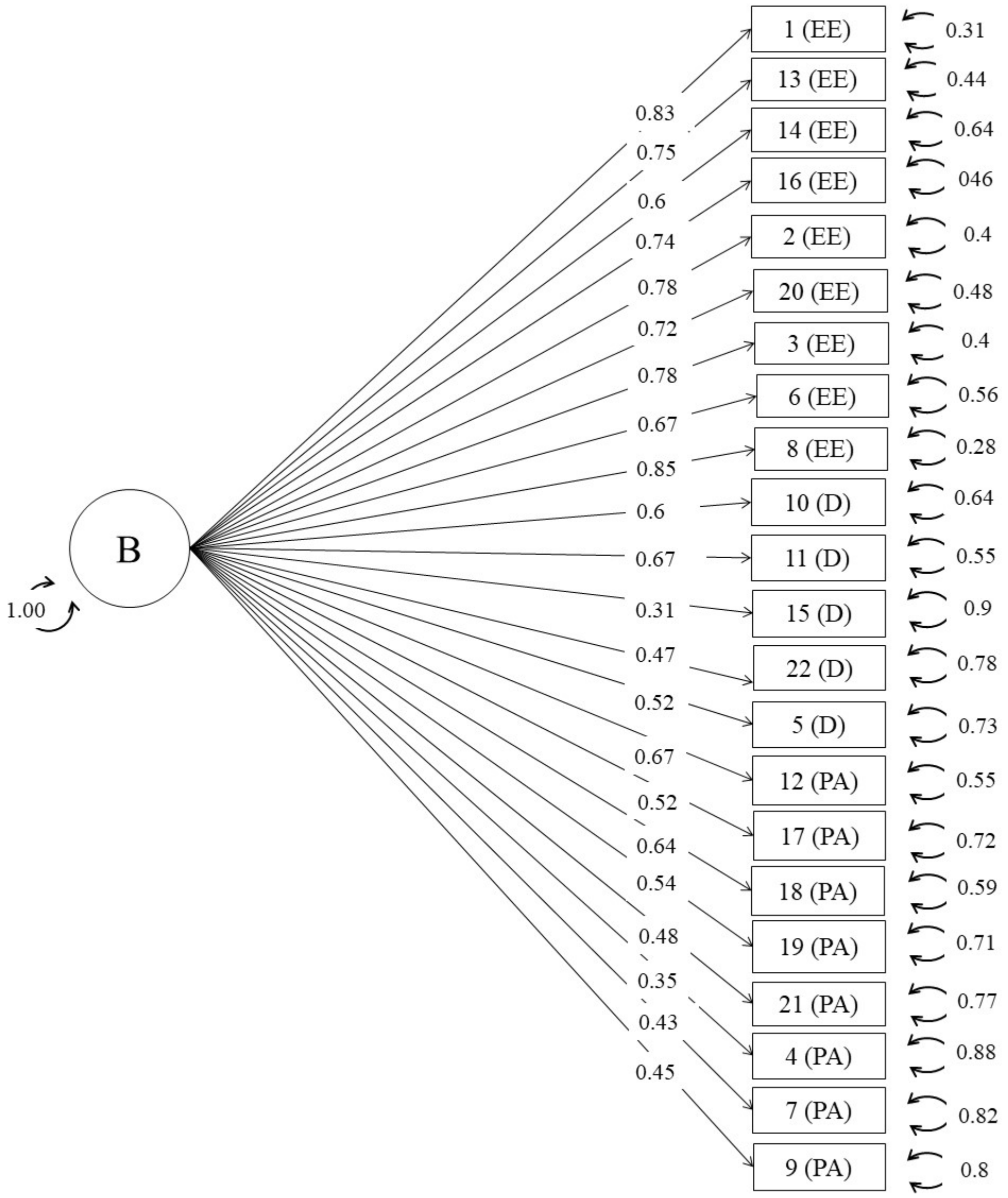

Note. Standardized solution for the bifactor model fitted to the 9 inter-item correlation matrices of the included studies.

Figure S4

Solution for the three-factor τ-equivalent model with two-stage MASEM

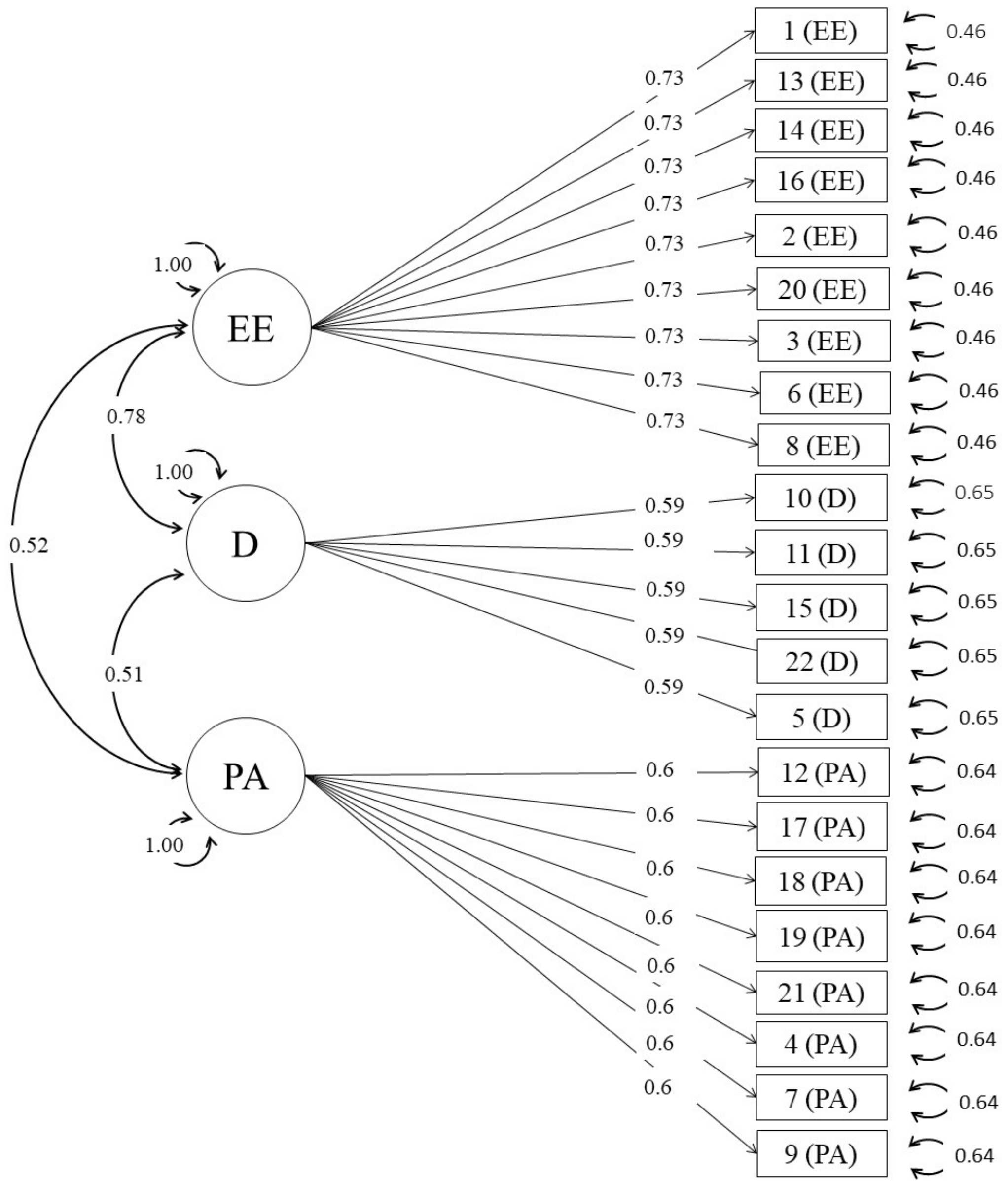

Note. Standardized solution for the bifactor model fitted to the 9 inter-item correlation matrices of the included studies.

Figure S5

Solution for the three-factor congeneric model with two-stage MASEM

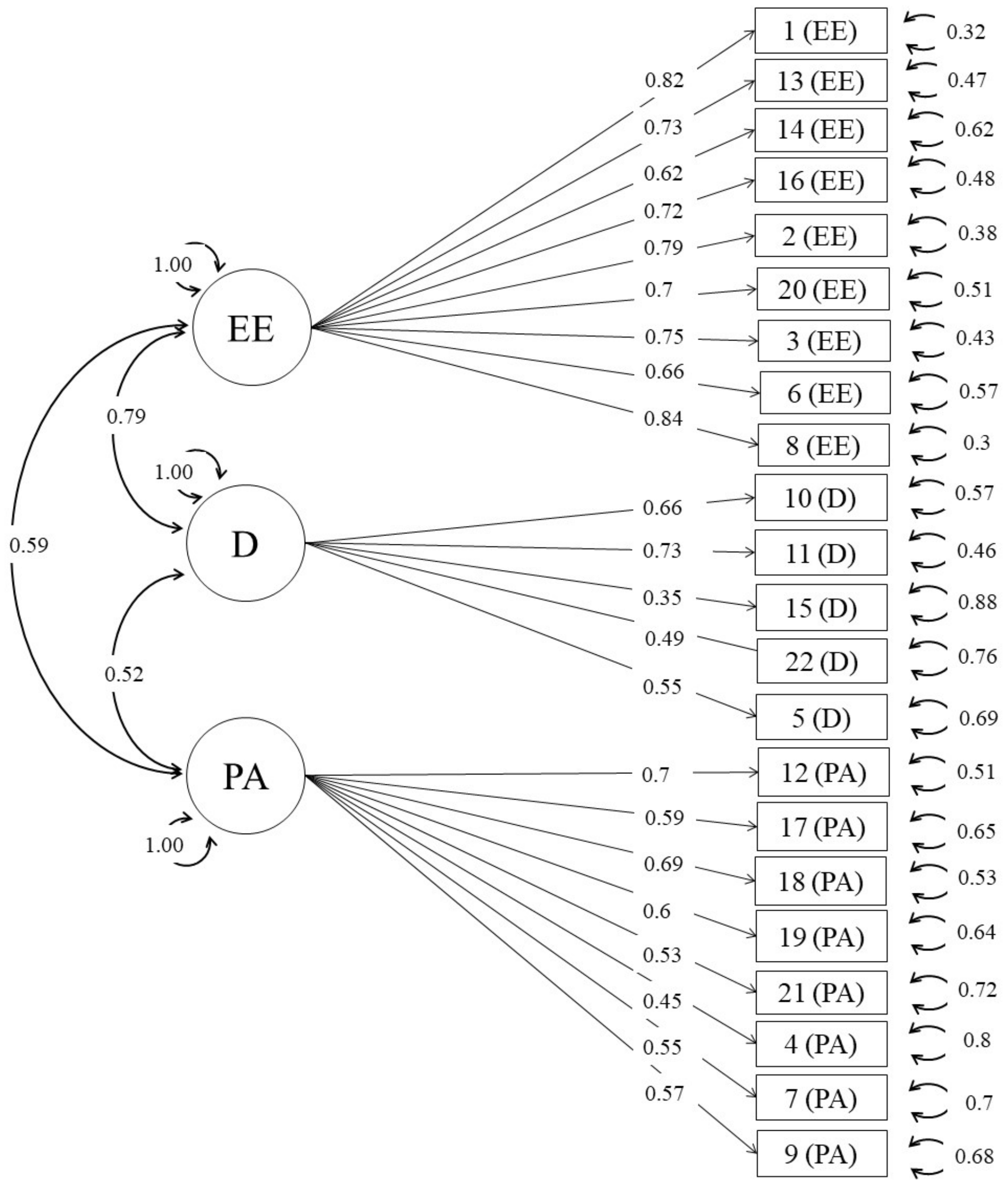

Note. Standardized solution for the bifactor model fitted to the 9 inter-item correlation matrices of the included studies.

Figure S6

Solution for the one-factor τ-equivalent model with one-stage MASEM

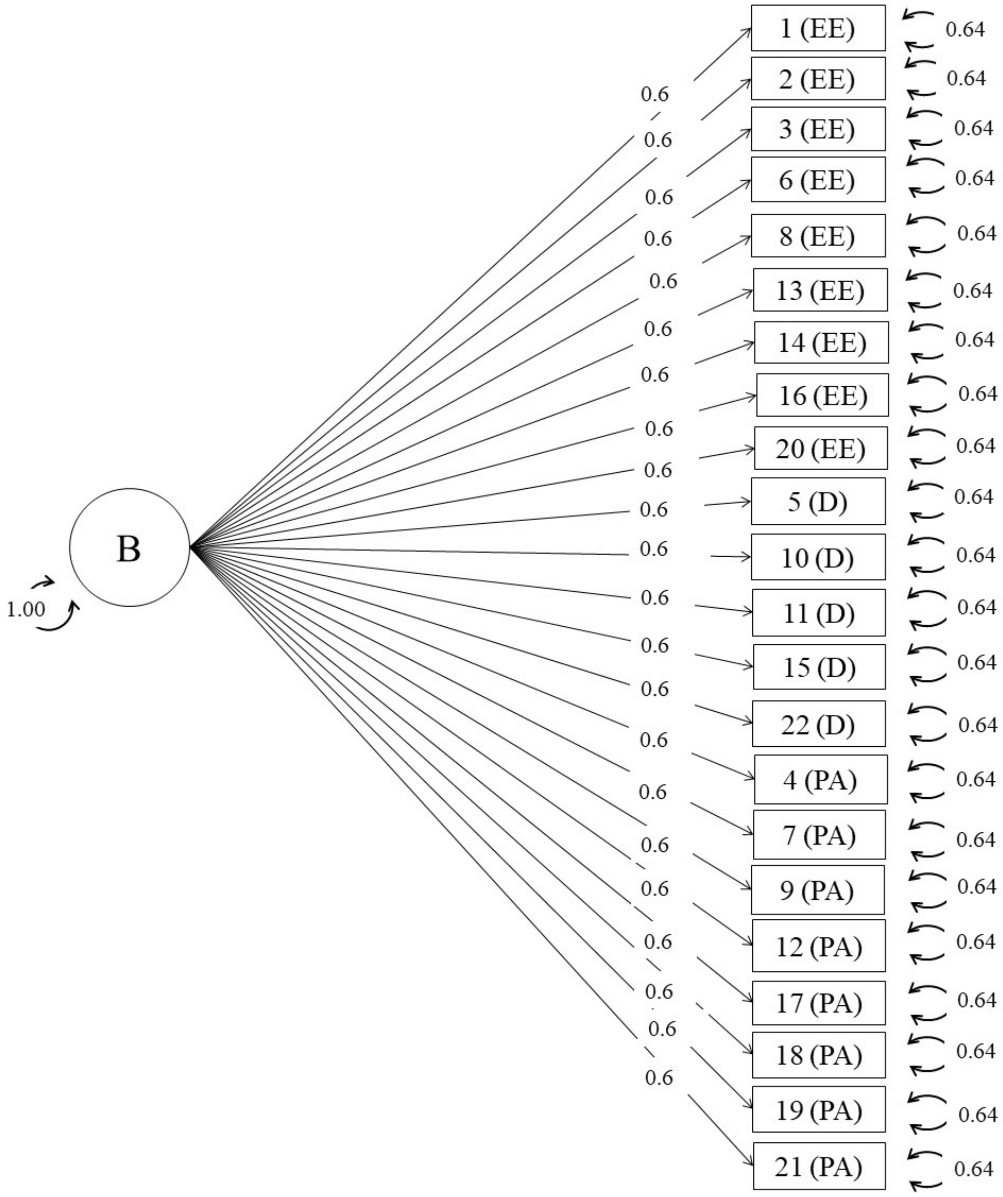

Note. Standardized solution for the one-factor τ-equivalent model fitted to the 9 inter-item correlation matrices of the included studies.

Figure S7

Solution for the one-factor congeneric model with one-stage MASEM

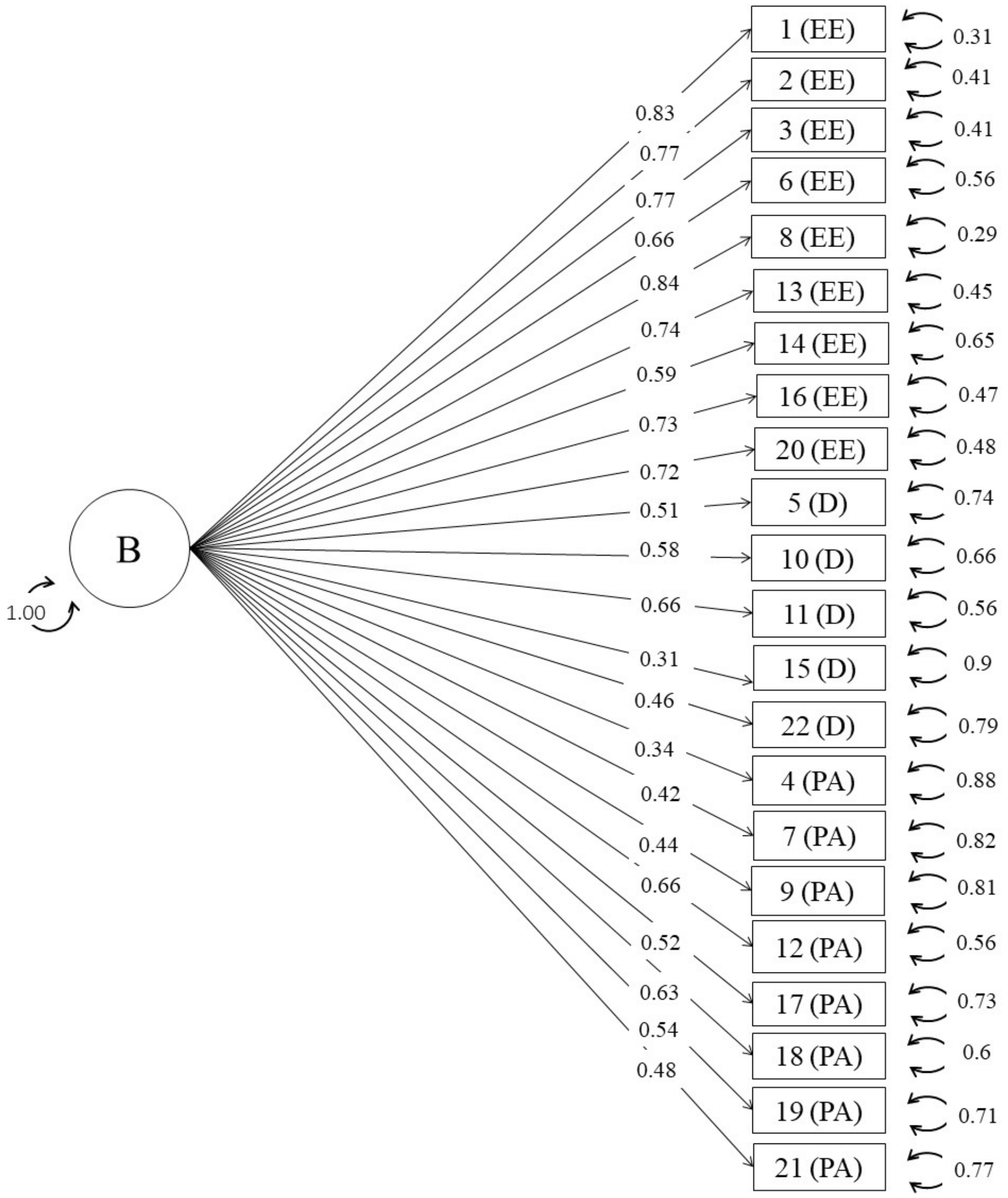

Note. Standardized solution for the one-factor congeneric model fitted to the 9 inter-item correlation matrices of the included studies.

Figure S8

Solution for the three-factor τ-equivalent model with one-stage MASEM

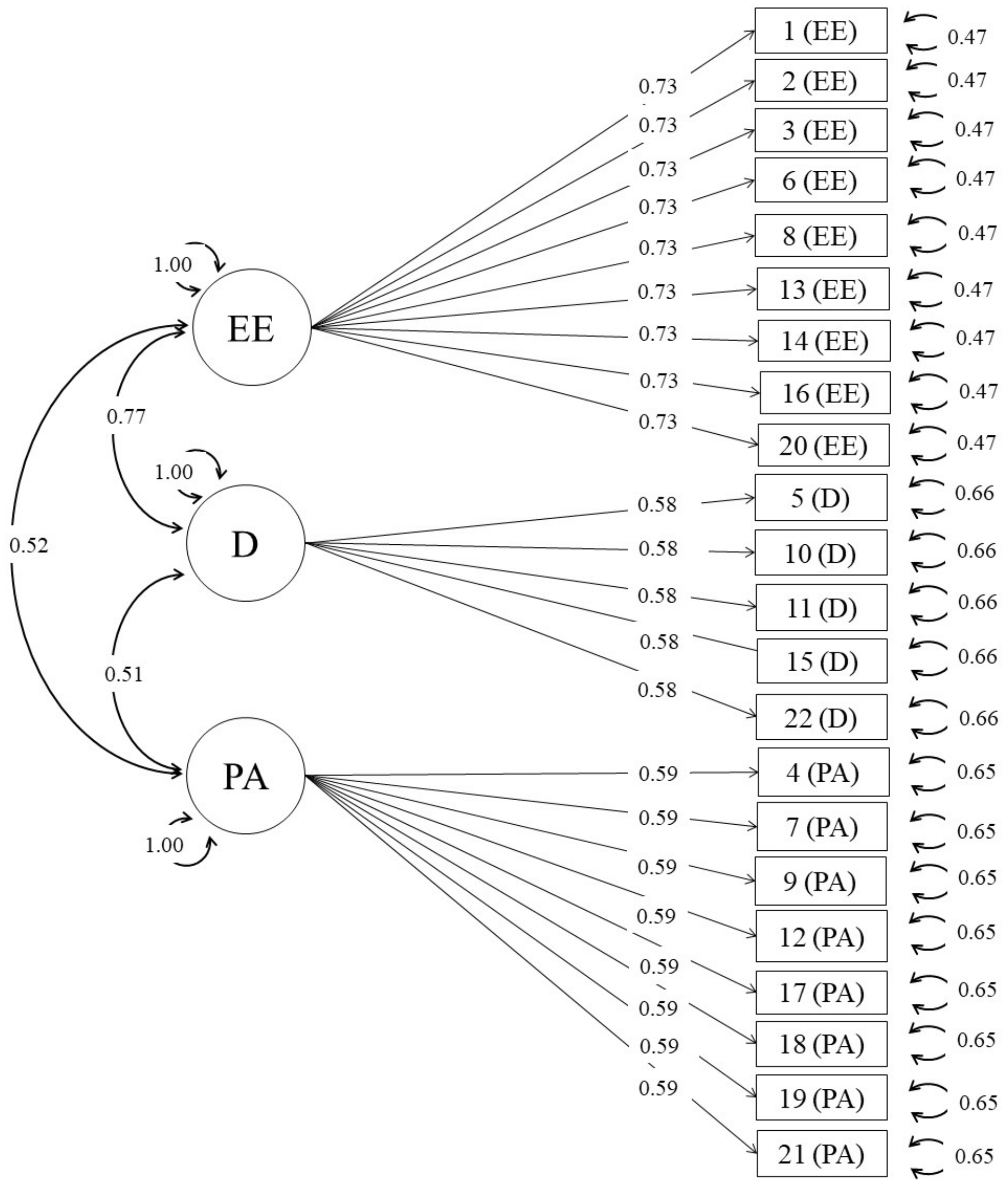

Note. Standardized solution for the bifactor model fitted to the 9 inter-item correlation matrices of the included studies.

Figure S9

Solution for the three-factor congeneric model with one-stage MASEM

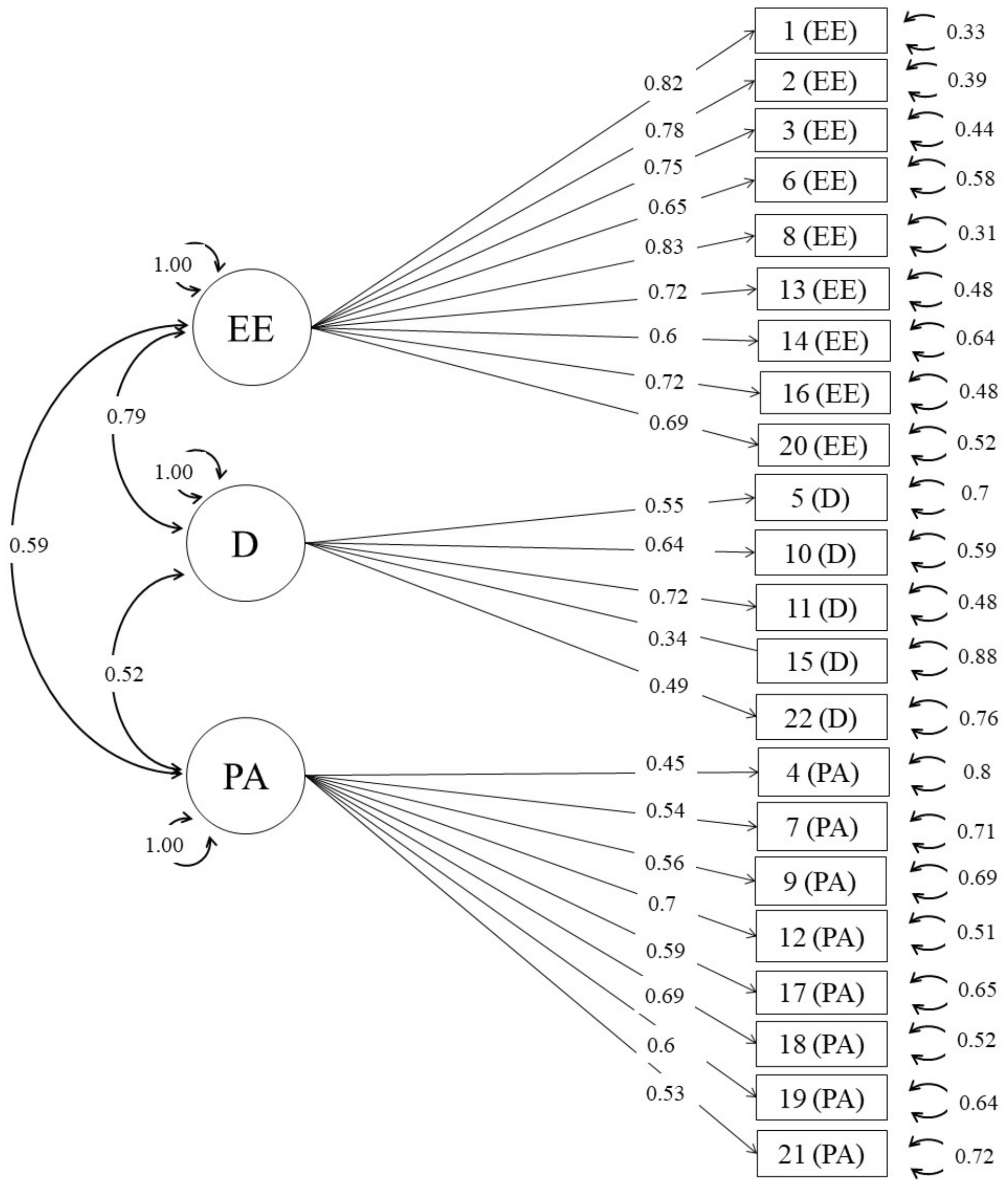

Note. Standardized solution for the bifactor model fitted to the 9 inter-item correlation matrices of the included studies.

References of the new studies included in the present meta-analysis

Aboagye, M., Qin, J., Qayyum, A., Antwi, C., Jababu, Y., & Affum-Osei, E. (2018). Teacher burnout in pre-schools: A cross-cultural factorial validity, measurement invariance and latent mean comparison of the Maslach Burnout Inventory, Educators Survey (MBI-ES). *Children and Youth Services Review*, *94*, 186–197. https://doi.org/10.1016/j.childyouth.2018.09.041

Al Mutair, A., Al Mutairi, A., Chagla, H., Alawam, K., Alsalman, K., & Ali, A. (2020). Examining and Adapting the Psychometric Properties of the Maslach Burnout Inventory-Health Services Survey (MBI-HSS) among Healthcare Professionals. A*pplied Sciences-Basel*, *10*(5). https://doi.org/10.3390/app10051890

Bria, M., Ratiu, L., & Baban, A. (2010). The burnout-engagement relationship: The factorial validity of the Maslach burnout inventory for human services and the Utrecht work engagement scale. *Psychology & Health*, *25*, 165–166. https://doi.org/10.1111/j.1467-9450.2009.00770.x

Canadas-de La Fuente, G., San Luis, C., Lozano, L., Vargas, C., Garcia, I., & de la Fuente, E. (2014). Evidence for factorial validity of Maslach Burnout Inventory and burnout levels among health workers. *Revista Latinoamericana de Psicologia*, *46*(1), 44–52. https://doi.org/10.1016/S0120-0534(14)70005-6

Doherty, A., Mallett, J., Leiter, M., & McFadden, P. (2021). Measuring Burnout in Social Work Factorial Validity of the Maslach Burnout Inventory—Human Services Survey. *European Journal of Psychological Assessment*, *37*(1), 6–14. https://doi.org/10.1027/1015-5759/a000568

Garcia, R., Sangregorio, M., & Sanchez, M. (2019). Factorial Validity of the Maslach Burnout Inventory-Human Services Survey (MBI-HSS) in a Sample of Spanish Social Workers. *Journal of Social Service Research*, *45*(2), 207–219. https://doi.org/10.1080/01488376.2018.1480549

Gonzalez-Rodriguez, R., Alonso, J., Verde-Diego, C., & Padin, P. (2022). Psychometric properties of the Maslach Burnout Inventory—Human Services in Social Work professionals in Spain. *Health & Social Care in The Community*, *30*(3), 949–956. https://doi.org/10.1111/hsc.13256

Jaramillo, S., Moreno, S., & Rodriguez, V. (2017). Confirmatory factor analysis of the MBI in Colombian parents of children with trisomy 21. *Testing, Psychometrics, Methodology in Applied Psychology*, *24*, 285–294. https://doi.org/10.4473/TPM24.2.7

Kim, J., Koh, S., Kang, J., Kim, C., & Park, K. (2017). Burnout in Korean Hemato-oncologists: Main findings and validity of the Maslach Burnout Inventory. *Annals of Oncology*, *28*, 103–103. https://doi.org/10.1093/annonc/mdx621.034

Lin, C., Alimoradi, Z., Griffiths, M., & Pakpour, A. (2022). Psychometric properties of the Maslach Burnout Inventory for Medical Personnel (MBI-HSS-MP). *Heliyon*, *8*(2). https://doi.org/10.1016/j.heliyon.2022.e08868

Loera, B., Converso, D., & Viotti, S. (2014). Evaluating the Psychometric Properties of the Maslach Burnout Inventory-Human Services Survey (MBI-HSS) among Italian Nurses: How Many Factors Must a Researcher Consider? *Plos One*, *9*(12). https://doi.org/10.1371/journal.pone.0114987

Moalemi, S., Kavosi, Z., Beygi, N., Deghan, A., Karimi, A., & Parvizi, M. (2018). Evaluation of the Persian Version of Maslach Burnout Inventory-Human Services Survey among Iranian Nurses: Validity and Reliability. *Galen Medical Journal*, *7*(1). https://doi.org/10.22086/gmj.v0i0.995

Ogunsuji, O., Ogundipe, H., Adebayo, O., Oladehin, T., Oiwoh, S., Obafemi, O., Soneye, O., Agaja, O., Uyilawa, O., Efuntoye, O., Alatishe, T., Williams, A., Ilesanmi, O., & Atilola, O. (2022). Internal Reliability and Validity of Copenhagen Burnout Inventory and Oldenburg Burnout Inventory Compared with Maslach Burnout Inventory among Nigerian Resident

Doctors: A Pilot Study. *Dubai Medical Journal*, *5*(2), 89–95. https://doi.org/10.1159/000521376

Olivares-Faúndez, V., Mena-Miranda, L., Macía-Sepúlveda, F., & Jélvez-Wilke, C. (2014). Validez factorial del Maslach Burnout Inventory Human Services (MBI-HSS) en profesionales chilenos. *Universitas Psychologica*, *13*(1), 145–160. https://doi.org/10.11144/Javeriana.UPSY13-1.vfmb

Pisanti, R., Lombardo, C., Lucidi, F., Violani, C., & Lazzari, D. (2013). Psychometric properties of the Maslach Burnout Inventory for Human Services among Italian nurses: A test of alternative models. *Journal of Advanced Nursing*, *69*(3), 697–707. https://doi.org/10.1111/j.1365-2648.2012.06114.x

Slabsinskiene, E., Gorelik, A., Vasiliauskiene, I., Kavaliauskiene, A., & Zaborskis, A. (2020). Factorial Validity and Variance of the Maslach Burnout Inventory between Demographic and Workload Groups among Dentists of Lithuania. *International Journal of Environmental Research and Public Health*, *17*(24). https://doi.org/10.3390/ijerph17249154

Szigeti, R., Balazs, N., Bikfalvi, R., & Urban, R. (2017). Burnout and depressive symptoms in teachers: Factor structure and construct validity of the Maslach Burnout inventory-educators survey among elementary and secondary school teachers in Hungary. *Stress and Health*, *33*(5), 530–539. https://doi.org/10.1002/smi.2737

Trigo, T., de Freitas, C., Wang, Y., Ribeiro, F., de Lucia, M., Siqueira, J., Iosifescu, D., Hallak, J., & Fraguas, R. (2018). The Influence of Depression on the Psychometric Properties of the Maslach Burnout Inventory-Human Services Survey: A Cross-Sectional Study With Nursing Assistants. *Frontiers in Psychiatry*, *9*. https://doi.org/10.3389/fpsyt.2018.00695

Valente, M., Wang, Y., & Menezes, P. (2018). Structural validity of the Maslach Burnout Inventory and influence of depressive symptoms in banking workplace: Unfastening the

occupational conundrum. *Psychiatry Research*, *267*, 168–174. https://doi.org/10.1016/j.psychres.2018.05.069

Vazquez, J., Castillo, G., & Garcia, S. (2021). Burnout in Police Officers from Northern Mexico: A Validity Study of the Maslach Burnout Inventory. *Journal of Police and Criminal Psychology*, *36*(3), 558–567. https://doi.org/10.1007/s11896-021-09452-z

Vukmirovic, M., Rajovic, N., Pavlovic, V., Masic, S., Mirkovic, M., Tasic, R., Randjelovic, S., Mostic, D., Velickovic, I., Nestorovic, E., Milcanovic, P., Stanisavljevic, D., & Milic, N. (2020). The Burnout Syndrome in Medical Academia: Psychometric Properties of the Serbian Version of the Maslach Burnout Inventory-Educators Survey. *International Journal of Environmental Research and Public Health*, *17*(16). https://doi.org/10.3390/ijerph17165658
